# Shifting paradigm of maternal and perinatal death review system in Bangladesh: A real time approach to address sustainable developmental goal 3 by 2030

**DOI:** 10.12688/f1000research.11758.1

**Published:** 2017-07-12

**Authors:** Animesh Biswas

**Affiliations:** 1 Reproductive and Child Health, Centre for Injury Prevention and Research Bangladesh (CIPRB), Dhaka, Bangladesh

**Keywords:** Maternal death, neonatal death, death review system, Bangladesh

## Abstract

Recently, Bangladesh has made remarkable progress in reducing maternal and neonatal morality, even though the millennium developmental goal to reduce maternal and neonatal mortality was not achieved. Sustainable Developmental Goal (SDG) 3 has already been set for a new target to reduce maternal and neonatal deaths by 2030. The country takes this timely initiative to introduce a maternal and perinatal death review system. This review will discuss the shifting paradigm of the maternal and perinatal death review system in Bangladesh and its challenges in reaching the SDG on time. This review uses existing literature on the maternal and perinatal death review system in Bangladesh, and other systems in similar settings, as well as reports, case studies, news, government letters and meeting minutes.

Bangladesh introduced the maternal and perinatal death review system in 2010. Prior to this there was no such comprehensive death review system practiced in Bangladesh. The system was established within the government health system and has brought about positive effects and outcomes. Therefore, the Ministry of Health and Family Welfare of Bangladesh gradually scaled up the maternal and perinatal death review system nationwide in 2016 within the government health system. The present death review system highlighted real-time data use, using the district health information software(DHIS-2). Health mangers are able to take remedial action plans and implement strategies based on findings in DHIS-2. Therefore, effective utilization of data can play a pivotal role in the reduction of maternal and perinatal deaths in Bangladesh.

Overall, the maternal and perinatal death review system provides a great opportunity to achieve the SDG 3 on time. However, the system needs continuous monitoring at different levels to ensure its quality and validity of information, as well as effective utilization of findings for planning and implementation under a measureable accountability framework.

## Introduction

Death review systems for maternal deaths have been performed both in developed and developing countries for many years ^1^. The majority of the death reviews focus on maternal death at the facility level ^2– 16^, but there are small number of community death review systems trialled in countries with high maternal deaths ^17, 18^. In Bangladesh, a comprehensive death review system for maternal death was not functional in the health system until 2010. In addition, adequate registration and notification of deaths was lacking until 2010. The Bangladesh maternal mortality survey in 2001 showed that the maternal mortality ratio is 320 per 100,000 live births ^19^, and the millennium developmental goal (MDG) was set to reduce this to 143 per 100,000 live births by 2015 ^20^. Likewise, neonatal deaths were also aimed to be reduced by two-thirds in the same time frame. Considering these challenges and needs, the country initiated and established a maternal and neonatal death review system in 2010 to address the MDGs ^21^. The initial death review system ran for a year in a district of Bangladesh and showed some positive results ^21^. In the meantime, the results of the Bangladesh maternal mortality survey 2010 were published in 2011, and clearly showed a progressive reduction in maternal mortality - 194 per 100,000 livebirths ^22^. Although the annual death reduction rate did not achieve the target, the country started to use death review system data at a local level for the improvement of the overall situation. Subsequently, the country gradually expanded the death review into 10 districts by 2013, which covered approximately 20 million of the general population. Although the countdown2015 report mentioned that Bangladesh is one of nine countries with good progress in achieving the maternal death target by 2015 ^20^, the United Nations (UNs) report estimated that the maternal mortality ratio of Bangladesh in 2015 was 176 per 100,00 livebirths ^23^. Moreover, Bangladesh demographic and health survey report of 2014 stated that the neonatal mortality rate is 28 per 1000 live births ^24^.

The new sustainable development goal (SDG) 3 has set a universal target, which means Bangladesh has to reduce maternal mortality to less than 70 per 100,00 live births, as well as neonatal deaths to <12 per 1000 live births by 2030 ^1^. Considering the present targets, the Ministry of Health and Family Welfare (MoH&FW) of Bangladesh has put forward the maternal and perinatal death review system for national scale up in 2016. The country has revised the maternal and perinatal death review system to be more action oriented and named this as Maternal and Perinatal Death Surveillance and Response (MPDSR).

This reviewed discusses the shifting paradigm of the Bangladesh maternal and neonatal death review system in the last seven years and the effects of the death review system in the country, and its facilitation for reaching the SDG3 target. This review focusses on the most recent articles, news and reports published on the maternal and perinatal death review system in Bangladesh, as well as related articles in low income countries in a similar context. Google scholar and MEDLINE/PubMed were used to find suitable articles, and full text of articles were preferred to review. A total number of nine articles relating to the maternal and perinatal death review in Bangladesh were found in full text. Moreover, the study also reviewed available reports, case studies, government letters, meeting minutes, web articles and thesis papers relating to the topic.

## Inception of the system in Bangladesh

A maternal and perinatal death review explores medical and social causes related to maternal and neonatal deaths though a systematic process. Bangladesh initiated this type of intervention initially in one district named Thakurgaon with a population of around 1.4 million in 2010 ^21^. The system was run by the government though the Directorate General of Health Services (DGHS) in collaboration with Directorate General of Family Planning (DGFP) of the MoH&FW. The initiative was under a partnership of the government and UN maternal and neonatal health initiatives. The death review was funded by UNICEF and Bangladesh, initially through DFID, UK Aid and then Global Affairs Canada; technical implementation support was given by a national non-government organization, Centre for Injury Prevention and Research Bangladesh (CIPRB) under a partnership with UNICEF ^21^.

Previously, there were no structured death review tools used widely in the country that provided evidence-based data for preparing action plans at different levels to reduce maternal and neonatal deaths in Bangladesh. Therefore, the country adopted its death review tool considering existing tools from various sources, such as Bangladesh maternal mortality survey, hospital base facility death review tool used by DGFP, World Health Organization (WHO) verbal autopsy tool, and tools developed by the Obstetric and Gynaecological Society of Bangladesh. A national technical group was formed to review all existing tools to prepare a simpler version for the country to use. Technical persons from DGHS and DGFP, professional experts, UNs, researchers and public health experts worked together to finalize these tools and guidelines under a participatory process. Next, these were endorsed by the government to use in the targeted district ^25^.

The guiding principal of the WHO ‘beyond the numbers’ for the maternal death audit was followed, where the entire system maintained confidentiality, non-blaming, anonymity and a non-punitive approach ^26^.

The system highlighted both community and facility death notifications of maternal deaths, and neonatal deaths and stillbirths though the government health system. Subsequently, for community deaths, each of the maternal deaths, neonatal death and stillbirths were reviewed. In addition, social intervention through social interaction with the community to discuss preventable future deaths was also performed, called ‘social autopsy’. Maternal and perinatal death review findings were used for local level planning, and implementation of those action plans was performed by health managers to reduce such preventable deaths ^25^. Death review findings were discussed and analysed at death review committees at the upazila and district level. A number of local action plans were taken and implemented using the findings from the death review committee meetings ^21, 25^.

## Scale up of the system

In following years, the death review system gradually expanded from four districts in 2011 to ten districts by 2013 ^27^. Moreover, at the beginning of 2015, Save the Children supported the government in introducing Maternal and Perinatal Death Review (MPDR) in four districts where MaMoni Health System Strengthening Projects were running ^28^. Overall, by 2015, 14 districts were under the coverage of MPDR, and the surveillance system covered approximately 28 million of the general population.

In 2015, the government took the initiative countrywide for its national scale-up, considering its need and importance to address the quality of care on maternal and neonatal health, as well as to reach the SDG3 in time. As part of this initiative, the Health Economics Unit of the Quality Improvement Secretariat of the MoH&FW took the initiative to update the national guideline and training manual for health care providers ^29– 33^. Three technical working groups were formed at a national level ^30^ and followed the maternal death surveillance and response (MDSR) framework developed by the WHO in 2013, in order to fit the revised version of death review system ^34^. The previous version of the death review system did not focus on ‘response’; therefore, this component was embedded within the existing MPDR. The updated version of the death review system highlighted death ‘surveillance and response’ in the conceptual framework, and was renamed Maternal and Perinatal Death Surveillance and Response (MPDSR). New revised version of National guideline, ToT manual and tools were approved by the ministry of health and family welfare for country use. The revised, simplified maternal and neonatal death review tools allow field level government health workers to complete the review more easily. MPDSR also highlights the integration of the quality improvement committee at sub-district, district, divisional and national levels to review the progress and respond to findings. In addition, facility-based MPDSR sub-committee at various levels was included to review facility deaths, in order to improve quality of care in facilities. Furthermore, the new system has introduced a focal person for MPDSR from the health department at sub-district, district, division and national levels to closely monitor and supervise MPDSR activities, including reporting to the quality improvement committees ^34^.

## Using District Health Information Software 2

The system has been further enriched by integrating the death review data in the District Health Information Software 2 (DHIS-2) of the Health Management Information System (HMIS) of DGHS. This has strengthened the overall health system by effective reporting of data online. Therefore, all death notification data entered in the DHIS-2 by a community health care provider, who works in a community clinic (small unit of outpatient service for maternal and neonatal health at the village level run by the government), can be seen at any time using the DHIS-2 platform by the health managers of different levels for immediate planning and interventions based on the data ^35^. Similarly, cause analysis of community maternal and neonatal deaths, including ICD-10 codes, are assigned at a divisional level by professional experts of medical college hospitals (obstetricians & gynaecologists and neonatologists). The Quality Improvement Secretariat of the MoH&FW organizes capacity development training for professional experts performing manual analysis on possible medical causes, including factors associated with death using a community verbal autopsy form ^36^. Final causes of death are inserted into the DHIS-2 at the divisional level in a periodic basis. This creates a unique opportunity for the upazilas and district level health managers, as well as for national level policy makers, planners, researchers, developmental partners and related stakeholders to understand and track the maternal and neonatal death situation in Bangladesh.

## Impact of the system

The development of the death review system for maternal and neonatal death has had a positive effect on the improvement of overall maternal and neonatal health, including playing a pivotal role in the reduction of a significant number of deaths. Various components of the system have been evaluated to moderate its effects ^37^. Notifications of maternal and neonatal deaths at the community level allows the deaths from the whole community to be captured, including under-privileged areas and hard to reach areas. Therefore, the maternal and neonatal mortality ratios that are calculated yearly in districts provide an accurate idea on the maternal and neonatal health situations in the particular areas. The notification system also highlights areas with a high amount of deaths. Specific interventions taken by local managers in death-dense areas can lead to a reduction in deaths in subsequent years ^38^. Another utilization of the death notification system is its incorporation with DHIS-2, which helps to visualise the live data online. In addition, local planning, such as using death spot maps at the sub-districts level, helps health managers to concentrate where interventions are needed ^39^.

Verbal autopsy in the death review system at the community level is used to explore medical causes, causes of delays and social factors associated with deaths reported by the MPDSR in Bangladesh ^40, 41^. Findings of verbal autopsies are used by local health managers for effective planning and reduction of such deaths in the future, causing improvements in 1 ^st^ delay (decision making) and 2 ^nd^ delays (transferring to referral centre) and improvement in referrals to specific facilities ^42^.

Facility death review of maternal and neonatal deaths can also be used to explore the causes of deaths in the facilities, as well as factors associated with the deaths, including gaps and challenges in the facility, which can then be overcome. The district uses these findings for effective planning and can intervene accordingly. For example, in one district, the provision of blood donors for an emergency supply of blood for mothers with postpartum haemorrhage (PPH) reduced the amount of deaths due to PPH ^43^.

Social autopsy in the death review system has allowed communities to engage in dialog on the causes of the deaths, explore social stigmas and barriers, and try to explore possible solutions for the prevention of preventable future deaths under an effective and harmonized dialog between the community and health care providers who facilitate the social autopsy interviews ^25, 44^. It has been observed over the years that social autopsy is able to explore social causes behind deaths ^45^. Community interaction enhances the ownership of the community, allows understanding of their own problems, and empowers the community to think and take appropriate action. Representative publicly-elected community leaders at the village level can change social barriers to prevent unwanted deaths, minimize delays and influence pregnant mothers to uptake care at government health facilities ^46, 47^.

## A unique approach

The MPDSR in Bangladesh is unique, as it not only includes death surveillance and response, but also covers community and facility maternal and newborn deaths together. In addition, the MPDSR captures stillbirth data though death notification system. The system is a national programme of the government and is led by various sections of the government for its implementation within the MoH&FW, including DGHS, DGFP, Management Information System (MIS) and Health Economic Unit (HEU), along with support of developmental partners, professional bodies and non-government organizations. The government health providers working in the health system are the key actors in the MPDSR roll out, which is sustainable and replicable in other countries with similar settings. Similarly, quality improvement committees at different level is the main platform for planning and implementation of various interventions. The data platform DHIS-2 is a live surveillance system and effective monitoring tool to see the progress of the country to reach the maternal and neonatal death reduction by 2030 as set out in SDG3 ^34^. Initial costing of the development phase of the death review system was found to be high; however, cost estimation has shown that field implementation of the system is much lower in order to sustain and run the system in the ongoing government health system ^48^.

South-East Asian countries, such as India, Pakistan, Sri Lanka, Nepal and the Maldives, have already implemented death reviews for maternal deaths. India has conducted maternal and perinatal death inquiry and response in a few districts with an aim to work on community deaths. However, this system does not explore facility deaths, or how best to use the outcome data ^17, 49^. A study conducted in India has calculated both community and facility deaths, but was restricted to maternal deaths only ^11^. By contrast, Pakistan has determined maternal death cause identification using verbal autopsy ^17^. A recent report by the WHO on MDSR highlighted the role of death surveillance and response in the reduction of maternal mortality in order to address the SDGs ^1^. It also focused on both community and facility maternal death review.

## Challenges ahead

The new approach of MPDSR in Bangladesh could be an evidence-based example for other low and middle income countries in achieving the best effects at a local level using a government health system, which has achieved functional planning and implementation of decisions based on death review findings. However, many countries around the world have reported that maternal death review results showed disparity between policy and practice; especially, effective coordination and planning requirement for a timely response remain a challenge ^4, 50, 51^.

Experiences of the early implementation of the Bangladesh maternal and perinatal death review from 2010 to 2015 has shown some strategic challenges. Previous studies mentioned that challenges still persist in capturing all deaths from the community, especially from pocket areas in hard to reach districts ^38, 39^. Ensuring the quality of data though conduction of verbal autopsy in the community and the best utilization of the findings at various level is also a challenge ^40– 42^. Moreover, social autopsy findings showed that social barriers are still a key issue in averting maternal and neonatal deaths ^45, 46^. It is recommended that social autopsy intervention in the community could play pivotal role in changing the beliefs and practices of the community to seek appropriate care during pregnancy, delivery and postpartum period. Social mobilization also influences social empowerment through engagement and social commitment; therefore, social autopsies in MPDSR have an enormous opportunity for use in other countries ^44^. At a facility level, limitation of proper documentation and record keeping has been identified as a key barrier to reviewing facility deaths ^43^.

## Conclusions

The shifting of Bangladesh from the MPDR to the MPDSR is a timely initiative to achieve the SDG3 in Bangladesh. A death notification system is able to capture all maternal deaths, neonatal deaths and stillbirths in the community and facility, while the death review for maternal and neonatal deaths explores the causes, gaps, and challenges that needed to be overcome. The quality improvement committees at different levels closely monitor the progress and intervene accordingly. At a local level, action plans use live data in the HMIS guide, which supports health managers and planners to make decisions and implement effective and focused interventions for achieving the SDG3 in Bangladesh by 2030.

## References

[1] World Health Organization: Time to respond: a report on the global implementation of maternal death surveillance and response (MDSR). World Health Organization.Maternal, newborn, child and adolescent health.2016 Reference Source

[2] Kongnyuy EJ Mlava G van den Broek N: Facility-based maternal death review in three districts in the central region of Malawi: an analysis of causes and characteristics of maternal deaths. *Womens Health Issues.* 2009;19(1):14–20. 10.1016/j.whi.2008.09.008 19111783

[3] Dumont A Tourigny C Fournier P: Improving obstetric care in low-resource settings: implementation of facility-based maternal death reviews in five pilot hospitals in Senegal. *Hum Resour Health.* 2009;7:61. 10.1186/1478-4491-7-61 19627605 PMC2728704

[4] Kongnyuy EJ van den Broek N: The difficulties of conducting maternal death reviews in Malawi. *BMC Pregnancy Childbirth.* 2008;8:42. 10.1186/1471-2393-8-42 18786267 PMC2546364

[5] Hofman JJ Mohammed H: Experiences with facility-based maternal death reviews in northern Nigeria. *Int J Gynecol Obstet.* 2014;126(2):111–4. 10.1016/j.ijgo.2014.02.014 24834852

[6] Osman H Campbell OM Sinno D: Facility-based audit of maternal mortality in Lebanon: a feasibility study. *Acta Obstet Gynecol Scand.* 2009;88(12):1338–44. 10.3109/00016340903318014 19900073

[7] Khanam RA Khan M Halim MA: Facility and Community Based Maternal Death Review in Bangladesh. *Bangladesh J Obstet Gynaecol.* 2009;24(1):18–21. 10.3329/bjog.v24i1.6322

[8] Pearson L deBernis L Shoo R: Maternal death review in Africa. *Int J Gynaecol Obstet.* 2009;106(1):89–94. 10.1016/j.ijgo.2009.04.009 19428010

[9] Oladapo OT Adetoro OO Fakeye O: National data system on near miss and maternal death: shifting from maternal risk to public health impact in Nigeria. *Reprod Health.* 2009;6:8. 10.1186/1742-4755-6-8 19508717 PMC2702364

[10] Olsen BE Hinderaker SG Bergsjø P: Causes and characteristics of maternal deaths in rural northern Tanzania. *Acta Obstet Gynecol Scand.* 2002;81(12):1101–9. 10.1034/j.1600-0412.2002.811202.x 12519105

[11] Jafarey SN Rizvi T Koblinsky M: Verbal autopsy of maternal deaths in two districts of Pakistan--filling information gaps. *J Health Popul Nutr.* 2009;27(2):170–83. 10.3329/jhpn.v27i2.3329 19489414 PMC2761775

[12] Dumont A Gaye A de Bernis L: Facility-based maternal death reviews: effects on maternal mortality in a district hospital in Senegal. *Bull World Health Organ.* 2006;84(3):218–24. 16583081 10.2471/blt.05.023903 PMC2627298

[13] Supratikto G Wirth ME Achadi E: A district-based audit of the causes and circumstances of maternal deaths in South Kalimantan, Indonesia. *Bull World Health Organ.* 2002;80(3):228–34. 11984609 PMC2567753

[14] Goswami D Rathore AM Batra S: Facility-based review of 296 maternal deaths at a tertiary centre in India: could they be prevented? *J Obstet Gynaecol Res.* 2013;39(12):1569–79. 10.1111/jog.12099 23875755

[15] Dumont A Gaye A De Bernis L: Lessons from the Field Facility-based maternal death reviews: effects on maternal mortality in a district hospital in Senegal.2006; 023903(05). Reference Source 10.2471/blt.05.023903 PMC2627298 16583081

[16] Pearson L deBernis L Shoo R: Maternal death review in Africa. *Int J Gynaecol Obstet.* 2009;106(1):89–94. 10.1016/j.ijgo.2009.04.009 19428010

[17] Dikid T Gupta M Kaur M: Maternal and perinatal death inquiry and response project implementation review in India. *J Obstet Gynaecol India.* 2013;63(2):101–7. 10.1007/s13224-012-0264-3 24431614 PMC3664695

[18] Bayley O Chapota H Kainja E: Community-linked maternal death review (CLMDR) to measure and prevent maternal mortality: a pilot study in rural Malawi. *BMJ Open.* 2015;5(4):e007753. 10.1136/bmjopen-2015-007753 25897028 PMC4410129

[19] National Institute of Population Research and Training (NIPORT), ORC Macro, Johns Hopkins University and ICDDR,B: Bangladesh Maternal Health Services and Maternal Mortality Survey 2001. Dhaka, Bangladesh and Calverton, Maryland (USA),2003 Reference Source

[20] El Arifeen S Hill K Ahsan KZ: Maternal mortality in Bangladesh: a Countdown to 2015 country case study. *Lancet.* 2014;384(9951):1366–74. 10.1016/S0140-6736(14)60955-7 24990814

[21] Biswas A Rahman F Halim A: Maternal and Neonatal Death Review (MNDR): A Useful Approach to Identifying Appropriate and Effective Maternal and Neonatal Health Initiatives in Bangladesh. *Health.* 2014;6:1669–79. 10.4236/health.2014.614198

[22] National Institute of Population Research and Training (NIPORT), MEASURE Evaluation, ICDDR,B: Bangladesh Maternal Mortality and Health Care Survey 2010. Summary of key findings and Implementation. Dhaka, Bangladesh,2012 Reference Source

[23] WHO, UNICEF, UNFPA: Trends in maternal mortality: 1990 to 2015. WHO, Sexual and reproductive health,2015 Reference Source

[24] National Institute of Population Research and Training (NIPORT), Mitra and Associates, ICF International: Bangladesh Demographic and Health Survey 2014. Dhaka, Bangladesh, and Rockville, Maryland, USA,2016 Reference Source

[25] Biswas A: Maternal, newborn, child and adolescent health Maternal and Perinatal Death Review (MPDR): Experiences in Bangladesh. World Health Organisation,2015;1–5, [cited 20167 Jan 19]. Reference Source

[26] Lewis G: Beyond the numbers: reviewing maternal deaths and complications to make pregnancy safer. *Br Med Bull.* 2003;67(1):27–37. 10.1093/bmb/ldg009 14711752

[27] Directorate general of Health Services (DGHS): MPDR newsletter. DGHS.2015; [Cited on 29 June 2017]. Reference Source

[28] Save the Children, Bangladesh: MaMoni Newsletter. Save the Children, Bangladesh,2015; [Cited on 29 June 2017]. Reference Source

[29] Biswas A: Scaling-up Maternal and Perinatal Death Reviews in Bangladesh. MDSR action network.2015; [Cited on 15 June 2017]. Reference Source

[30] Ministry of Health and Family Welfare, Bangladesh: QIS newsletter. Ministry of Health and Family Welfare, Bangladesh.2015; [Cited on 29 June 2017]. Reference Source

[31] Quality Improvement Secretariat, MoHFW: E-newsletter. Quality Improvement Secretariat, MoHFW.2016; [Cited on 29 June 2017]. Reference Source

[32] Mahmud R: Rolling out MPDSR across the country. MDSR action network.2016; [Cited on 15 June 2017]. Reference Source

[33] Mahmud R Biswas A: The roll out of MPDSR. Cited on 15 June 2017. Reference Source

[34] Ministry of Health and Family Welfare (MoHFW): National Guideline on MPDSR.2016; [cited 2017 Jan 28]. Reference Source

[35] Biswas A: Using eHealth to support MPDR: Early experiences from Bangladesh. MDSR Action Network,2015;6–8. Reference Source

[36] Quality Improvement Secretariat, MoHFW: E-newsletter. Quality Improvement Secretariat, MoHFW,2017; [Cited on 29 June 2017]. Reference Source

[37] Biswas A: Maternal and Neonatal Death Review System to Improve Maternal and Neonatal Health Care Services in Bangladesh. Örebro University,2015 Reference Source

[38] Biswas A Rahman F Eriksson C: Community Notification of Maternal, Neonatal Deaths and Still Births in Maternal and Neonatal Death Review (MNDR) System: Experiences in Bangladesh. *Health.* 2014;6(16):2218–26. 10.4236/health.2014.616257

[39] Biswas A: MDSR Action Network Mapping for action: Bangladesh. MDSR Action Network,2015;1–5, [cited 2017 Jan 27]. Reference Source

[40] Halim A Utz B Biswas A: Cause of and contributing factors to maternal deaths; a cross-sectional study using verbal autopsy in four districts in Bangladesh. *BJOG.* 2014;121(Suppl 4):86–94. 10.1111/1471-0528.13010 25236640

[41] Halim A Dewez JE Biswas A: When, Where, and Why Are Babies Dying? Neonatal Death Surveillance and Review in Bangladesh. *PLoS One.* 2016;11(8):e0159388. 10.1371/journal.pone.0159388 27478900 PMC4968790

[42] Biswas A Rahman F Halim A: Experiences of Community Verbal Autopsy in Maternal and Newborn Health of Bangladesh. *HealthMED.* 2015;9(8):329–38. Reference Source

[43] Biswas A Rahman F Eriksson C: Facility Death Review of Maternal and Neonatal Deaths in Bangladesh. *PLoS One.* 2015;10(11):e0141902. 10.1371/journal.pone.0141902 26540233 PMC4634754

[44] Biswas A: Social autopsy as an intervention tool in the community to prevent maternal and neonatal deaths: experiences from Bangladesh Social autopsy. *MDSR Action Network.* 2016;6–8, [cited 2017 Jan 24]. Reference Source

[45] Biswas A Halim MA Dalal K: Exploration of social factors associated to maternal deaths due to haemorrhage and convulsions: Analysis of 28 social autopsies in rural Bangladesh. *BMC Health Serv Res.* 2016;16(1):659. 10.1186/s12913-016-1912-6 27846877 PMC5111193

[46] Biswas A Rahman F Eriksson C: Social Autopsy of maternal, neonatal deaths and stillbirths in rural Bangladesh: qualitative exploration of its effect and community acceptance. *BMJ Open.* 2016;6(8):e010490. 10.1136/bmjopen-2015-010490 27554100 PMC5013352

[47] Mahmud R: Social autopsy triggers community response for averting maternal and neonatal death in Bangladesh. WHO,2016 Reference Source

[48] Biswas A Halim A Rahman F: The Economic Cost of Implementing Maternal and Neonatal Death Review in a District of Bangladesh. *J Public Health Res.* 2016;5(3):729. 10.4081/jphr.2016.729 28090477 PMC5228161

[49] UNICEF: Maternal and perinatal death inquiry and response. Empowering communities to avert maternal deaths in India, New Delhi: UNICEF,2009; [cited 2016 Dec 18]. Reference Source

[50] Singh S Murthy GV Thippaiah A: Community based maternal death review: lessons learned from ten districts in Andhra Pradesh, India. *Matern Child Health J.* 2015;19(7):1447–54. 10.1007/s10995-015-1678-1 25636651

[51] Armstrong CE Lange IL Magoma M: Strengths and weaknesses in the implementation of maternal and perinatal death reviews in Tanzania: perceptions, processes and practice. *Trop Med Int Heal.* 2014;19(9):1087–95. 10.1111/tmi.12353 25039579

