# Supplementary material for: Shifting paradigm of maternal and perinatal death review system in Bangladesh: A real time approach to address sustainable developmental goal 3 by 2030
Source: F1000Res. 2017 Jul 12;6:1120. [Version 1] doi: 10.12688/f1000research.11758.1 (PMC5585875; doi:10.12688/f1000research.11758.1)
Supplement: Peer review history

# Peer review: Referee response for version 1

**DOI:** 10.5256/f1000research.12701.r24173

**Published:** 2017-09-05

**Authors:** Olakunle Alonge

**Affiliations:** 1 Health Systems Program, Department of International Health, Johns Hopkins Bloomberg School of Public Health, Baltimore, MD, USA

This is a well-written review article and highlights an intervention for a very important public health problem - maternal deaths in low and middle income countries. The article is a very fine contribution to the literature.

 It could be further strengthened by providing specific recommendations on the way forward regarding maternal death notification, review and response in Bangladesh based on findings from other countries - perhaps, these recommendations could be presented in a table highlighting identified challenges and potential solutions. Similarly, a box or panel highlighting the essential components of the perinatal and maternal death review system in Bangladesh will also be useful for readers.

 The abstract could be rewritten as a summary of the literature review (highlighting the reason for the literature review, and briefly summarizing each segment of the paper). The abstract and paper could use further editing for typos.

I have read this submission. I believe that I have an appropriate level of expertise to confirm that it is of an acceptable scientific standard.

# Peer review: Referee response for version 1

**DOI:** 10.5256/f1000research.12701.r24174

**Published:** 2017-08-22

**Authors:** Mithila Faruque

**Affiliations:** 1 Department of Noncommunicable Diseases, Bangladesh University of Health Sciences (BUHS), Dhaka, Bangladesh

I have gone through the review article. To me, the author has taken a timely initiative to review on maternal and perinatal death review system and also he is experienced enough to write review on this topic. He has described the total MPDR system in Bangladesh from its initiation to scale up, benefits and challenges. But he didn’t mention in the whole paper what was the purpose of this review paper. Also some more comparison with the maternal and perinatal death review system of other countries should be added. The author could include a table showing the findings of different articles (used in reference) in short regarding MPDR, so that the readers can get a clear view of the review articles at a glance. Also the conclusion should be modified by adding the advantage of this review paper, not only of the death review system, I mean how this review paper will bring benefits to other researchers or how can it be used further in future.

 Also I have some corrections mentioned below (the correction lines are numbered as in the pdf file)

Introduction

- Last paragraph, line 1 - "This reviewed" needs to be corrected to "This review"
- Last paragraph, line 4 - " This review" needs to be corrected to "The review"
- Last paragraph line 9 - "full text of articles" needs to be corrected to "full text articles"

Inception of the system in Bangladesh

- First paragraph line 2 - "though" needs to be corrected to "through"
- Last paragraph line 3 - "though" needs to be corrected to "through"
- Last paragraph line 4 - "neonatal death" needs to be corrected to "neonatal deaths"

Scale up of the system

- Second paragraph line 18 - "ministry of health and family welfare" needs to be corrected to "Ministry of Health and Family Welfare"

A unique approach

- First paragraph line 4 - "though" needs to be corrected to "through"

I have read this submission. I believe that I have an appropriate level of expertise to confirm that it is of an acceptable scientific standard.
